# Cervical adenocarcinoma misdiagnosed as a nabothian cyst during pregnancy: A case report and review of the literatures

**DOI:** 10.1097/MD.0000000000042336

**Published:** 2025-05-30

**Authors:** Kyung Eun Lee, Ji Ae Kim, Min Jeong Kim, Hae Nam Lee, Jae Eun Shin

**Affiliations:** a Department of Obstetrics and Gynecology, College of Medicine, The Catholic University of Korea, Seoul, Republic of Korea.

**Keywords:** adenocarcinoma, case report, diagnostic error, pregnancy, uterine cervical neoplasm

## Abstract

**Rationale:**

The incidence of cervical adenocarcinoma is increasing, particularly in women of reproductive age, and presents a unique diagnostic and therapeutic challenge when encountered during pregnancy. Distinguishing malignant cervical lesions from benign entities such as nabothian cysts can be particularly difficult due to overlapping clinical and imaging features, leading to potential delays in diagnosis and management.

**Patient concerns:**

A 38-year-old multiparous woman at 7 weeks’ gestation was referred to a tertiary hospital for an abnormal Papanicolaou smear showing atypical glandular cells of undetermined significance and positive for high-risk human papillomavirus (HPV) type 18 infection. She was asymptomatic, with no vaginal bleeding, pelvic discomfort or other suggestive symptoms.

**Diagnoses:**

Initial imaging and colposcopic findings suggested a nabothian cyst. However, 4 weeks later, atypical cytological findings and HPV 18 positivity prompted further surveillance. At 26 weeks’ gestation, cervical biopsy confirmed moderately differentiated HPV-associated cervical adenocarcinoma. Magnetic resonance imaging staged the disease as International Federation of Gynecology and Obstetrics stage IB1.

**Interventions:**

After multidisciplinary consultation, the patient underwent classical cesarean section followed by type 3 radical hysterectomy, bilateral salpingectomy, pelvic lymphadenectomy and ovarian transposition at 29 weeks 6 days gestation. Magnesium sulfate and corticosteroids were administered for fetal neuroprotection and lung maturation.

**Outcomes:**

Pathology confirmed a 1.5 cm cervical adenocarcinoma without lymphovascular space invasion, parametrial extension or lymph node metastasis. The patient has remained disease-free for 4 years under regular oncological surveillance.

**Lessons:**

This case highlights the diagnostic complexity of cervical adenocarcinoma in pregnancy, particularly when lesions mimic benign cervical pathology. Persistent atypical cytology and high-risk HPV positivity warrant thorough evaluation, including colposcopic biopsy, despite pregnancy. A multidisciplinary approach is essential for optimal maternal and fetal outcomes.

## 1. Introduction

Cervical cancer was the eighth most common cancer among women in Korea in 2018, with a declining incidence over the past 20 years from 4488 cases in 1999 to 3500 in 2018.^[1]^ While the incidence of cervical squamous cell carcinoma declined from 13.27 to 6.16 per 100,000 between 1999 and 2018, the incidence of adenocarcinoma increased from 1.30 to 1.92 per 100,000 during the same period.^[1]^ Although malignancy coexisting with pregnancy is rare, the incidence of cervical cancer during pregnancy is increasing, posing a clinical challenge.^[2,3]^ We report a case of cervical adenocarcinoma that was misdiagnosed as a nabothian cyst during pregnancy.

## 2. Case presentation

**Figure 1.**
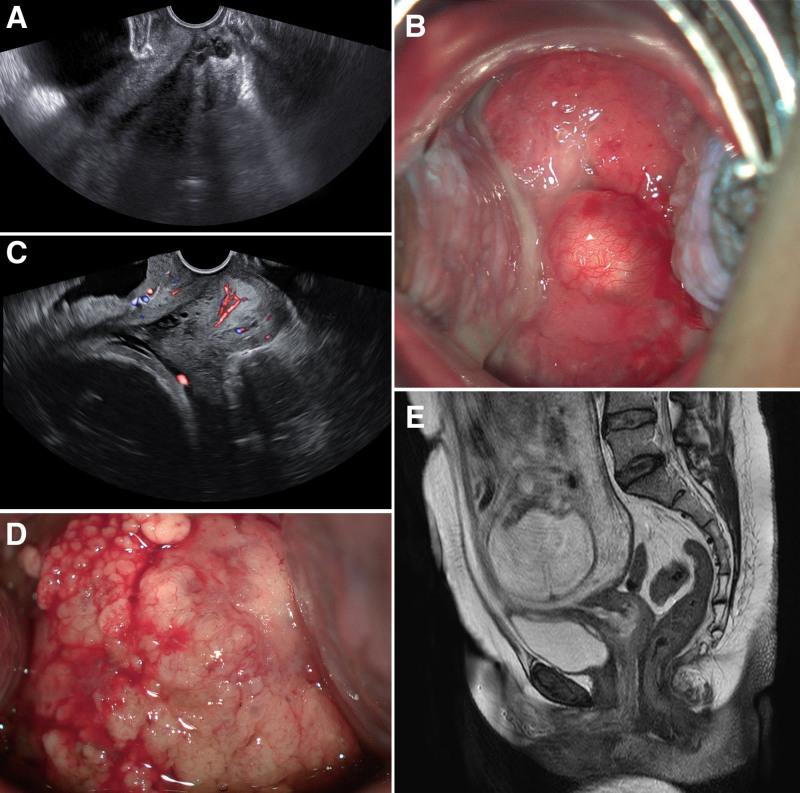
Imaging findings of the patient at 7 weeks and 26 weeks of gestation. (A) Pelvic ultrasound: a cystic lesion around 1.4 × 1.7 cm was seen in the posterior lip of cervix. (B) Colposcopy: a mass with a size of about 1 cm was seen at 6 O’ clock, mimicking a nabothian cyst at 7 weeks of gestation. (C) Colposcopy: 1.5 × 1.5 cm sized cervical mass with papillary nodules after rupture from cystic wall at posterior lip of cervix. (D) Pelvic ultrasound: 1.5 × 1.5 cm sized mass with plentiful vascularization was seen in the posterior lip of cervix. (E) Pelvic MRI without contrast: sagittal T2WI show 1.7 cm sized papillary mass with irregular margin of low signal intensity in the exocervix, at 26 weeks of gestation. MRI = magnetic resonance imaging.

**Figure 2.**
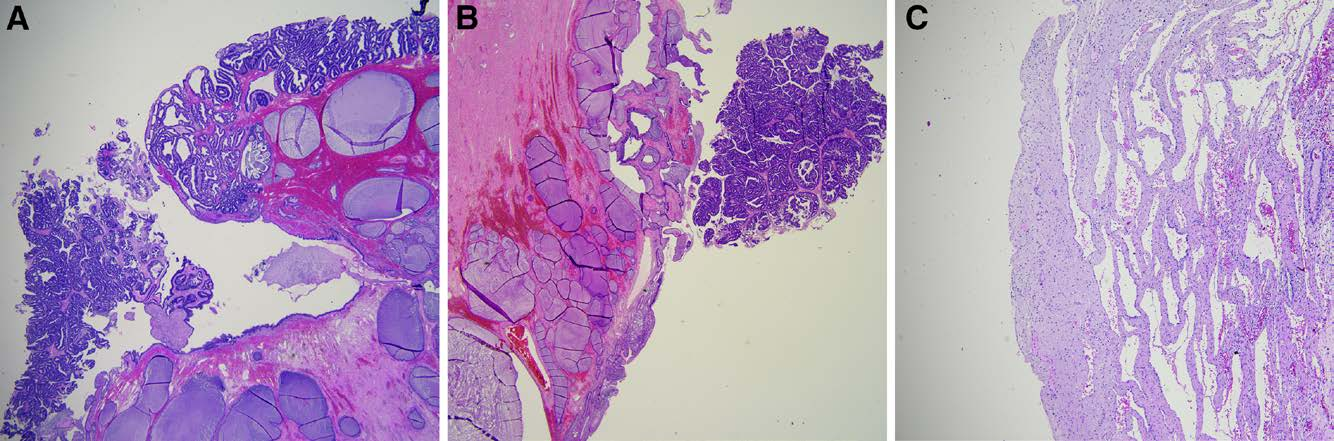
Histological images of the cervical adenocarcinoma. (A, B) The tumor showed a predominantly exophytic growth pattern near nabothian cyst. (C) Gestational change including decidua was seen in uterine corpus (using hematoxylin-eosin stain).

A 38-year-old multiparous woman was referred to our hospital because of abnormal Papanicolaou (PAP) smear test results, showing atypical glandular cells of undetermined significance and positive human papilloma virus (HPV) 18 infection at 7 weeks of gestation. The patient had no underlying diseases or abnormal symptoms such as abdominal pain, pelvic discomfort, or vaginal bleeding. Transvaginal ultrasonography revealed a well-defined cervical lesion without abnormal vascularity (Fig. 1A). A cervical mass was identified at the 6 o’clock direction, without cervical erosion on colposcopy (Fig. 1B). A PAP smear (liquid-based cytology using brush) and HPV testing were performed concurrently at the time of colposcopy, revealing reactive cellular changes with inflammation and HPV type 18 positivity. Based on these findings, along with benign-appearing features on ultrasound, the lesion was diagnosed as a nabothian cyst. At the 4-week follow-up, repeat assessments including ultrasound, colposcopy, and HPV testing revealed persistent cystic lesion on the cervical posterior lip, atypical glandular cells and HPV type 18 positivity. Based on multidisciplinary discussions between obstetricians and gynecologic oncologists, close surveillance was planned with a follow-up interval of 3 months. After 3 months, a transvaginal ultrasound scan showed a 1.5 cm solid mass in the cervix with plentiful vascularization (Fig. 1C). Colposcopy revealed a cervical mass with papillary nodules after rupture of the cystic wall at the posterior lip of the cervix (Fig. 1D). Therefore, we performed a colposcopic-directed cervical biopsy, and histological examination of the cervical sample revealed cervical adenocarcinoma diagnosed at 26 weeks of gestation. To evaluate the clinical stage of cervical cancer, gadolinium-free magnetic resonance imaging was performed, which showed a small papillary lesion approximately 1.7 cm in size suspected cervical cancer (Fig. 1E). The clinical stage of the patient was determined as stage IB1. She underwent a classical cesarean section, radical abdominal hysterectomy (type 3), bilateral salpingectomy, bilateral pelvic lymph node dissection (level 2), and bilateral ovarian transposition at 29 weeks 6days of gestation after administration of magnesium sulfate (MgSO_4_) fluid and corticosteroids to support fetal neurodevelopment and lung maturation. The pathologic report described a moderately differentiated HPV-associated cervical adenocarcinoma (1.5 × 0.7 cm) with involvement of the transformation zone, without lymphatic, perineural, parametrial, or vaginal invasion (Fig. 2A–C). No lymph node metastasis was observed. The patient has been under regular follow-up using enhanced abdominal and pelvic computed tomography with no recurrence of cervical cancer within 4 years.

## 3. Discussion

We report a case of cervical adenocarcinoma during pregnancy initially misdiagnosed as a nabothian cyst. Surgery was delayed following multidisciplinary discussions involving gynecologic oncologists, obstetricians, and neonatologists, allowing for fetal lung maturation and neurodevelopment. Once the treatment plan was established, the patient’s symptoms including vaginal bleeding, abdominal pain, and abnormal vaginal discharge were closely monitored.

Despite the overall decline in the number of squamous cell carcinoma cases, the incidence of cervical adenocarcinoma is increasing, particularly among younger women. Early-stage cervical cancer is increasingly observed in women in their 20s and 30s, which may contribute to the increasing prevalence of cervical cancer during pregnancy.^[4]^

Diagnosing cervical cancer during pregnancy is challenging due to several factors. Nabothian cysts, commonly found in the cervix, are generally considered incidental. Furthermore, signs of cervical cancer, such as vaginal bleeding and profuse discharge, may be confused with pregnancy-related symptoms. Detection is also challenging in early-stage malignancies, as these lesions are often asymptomatic. Physicians may also hesitate to perform invasive tests or to use advanced imaging studies during pregnancy. However, colposcopy-directed biopsy is a safe and reliable procedure during pregnancy and should be performed if malignancy is suspected.^[4]^

Previous studies have reported adenocarcinomas misdiagnosed as submucosal myomas^[5]^ or nabothian cysts.^[6]^ Transvaginal sonography and magnetic resonance imaging are the commonly used imaging modalities for cystic cervical lesions.^[7]^ Malignant tumors tend to invade deep into the cervical stroma, and a solid component surrounding or separating multiple cysts with significant vascularization can indicate malignancy. However, establishing an accurate diagnosis remains challenging.^[8]^ If the diagnosis is uncertain or cysts are deep-seated or large, excision for histopathologic evaluation is recommended.^[9]^

A multidisciplinary approach is essential for developing treatment strategies and providing counseling when cancer is diagnosed during pregnancy. A major challenge in cancer treatment during pregnancy is determining whether pregnancy should be maintained, depending on gestational age, malignancy type, metastasis, treatment options, and patient’s wishes.^[10]^ Previous studies have found no significant differences in maternal survival between patients who terminated or continued pregnancy (*P* = .964).^[2]^

## 4. Conclusion

It is challenging to diagnose cervical adenocarcinoma during pregnancy. Careful evaluation is essential if abnormal PAP findings or suspected benign conditions such as nabothian cysts are present. Biopsies should be performed if a malignancy is suspected.

## Author contributions

**Conceptualization:** Kyung Eun Lee, Jae Eun Shin.

**Data curation:** Kyung Eun Lee, Ji Ae Kim.

**Investigation:** Kyung Eun Lee, Ji Ae Kim.

**Project administration:** Jae Eun Shin.

**Supervision:** Jae Eun Shin.

**Validation:** Min Jeong Kim, Hae Nam Lee.

**Writing – original draft:** Kyung Eun Lee.

**Writing – review & editing:** Jae Eun Shin.
